# Study on Cytochrome P-450 Dependent Retinoic Acid Metabolism and its Inhibitors as Potential Agents for Cancer Therapy

**DOI:** 10.3797/scipharm.1106-18

**Published:** 2011-08-12

**Authors:** Mobasher Ahmad

**Affiliations:** University College of Pharmacy, University of the Punjab (Old Campus), the Mall, Lahore, Pakistan

**Keywords:** Retinoic Acid Metabolism blocking agents, RAMBAs, Cancer differentiation

## Abstract

The relative lack of clinical success with conventional anticancer agents may be due in part to the traditional concept of cancer being a biological state rather than a dynamic process. Redefining cancer as a dynamic disease commencing with carcinogenesis introduces the possibility of chemoprevention. Retinoids offer the promise of a therapeutic option based on differentiation of premalignant as well as malignant cells. Research to date has concentrated on the use of exogenous retinoids in cancer. Although this research continues with new retinoid derivatives, an alternative approach to overcoming the drawbacks associated with exogenous retinoids has been to increase the levels of endogenous retinoic acid (RA) by inhibiting the cytochrome P450- mediated catabolism of RA using a novel class of agents known as retinoic acid metabolism blocking agents (RAMBAs which increase the level of endogenous retinoic acid (RA) within the tumor cells by blocking their metabolism. This approach presents several theoretic advantages.

In the present study a wide range of established P-450 inhibitors has been screened to examine their inhibitory activity on *all-trans-*Retinoic acid (ATRA) metabolism. Forty-one known P450 inhibitors were tested for their inhibitory activity against RA metabolism. Most of them are nitrogen-containing compounds. The results showed that among these compounds only six compounds (*N*-benzyl-2-phenylethanamine, itraconazole, chlorpromazine, 5-chloro-1,3-benzoxazol-2-amine, proadifen and furazolidone) showed inhibition of RA metabolism which was > 50%. Ketoconazole and liarozole were also screened as standard potent inhibitors in the same system and gave 87.5% and 89% inhibition, respectively. The results indicate that mostly azoles with substituents in positions other than the 1-position on the ring are very weak inhibitors of RA metabolism. The most effective inhibitors (ketoconazole, itraconazole, bifonazole and clotrimazole) are 1-substituted and possess relatively large aromatic groups in the molecule. 1-Substituted imidazoles bind to cytochrome P-450 with a very high affinity but substitution in the other position of the imidazole decreases the binding affinity.

## Introduction

Although significant advances have been made in the treatment of some malignancies, the prognosis of patients with metastasis tumors remains poor. Differentiating agents redirect cells toward their normal phenotype and therefore may reverse or suppress evolving malignant lesions or prevent cancer invasion. In addition, they offer a potential alternative to the classic cytostatic drugs and indeed represent an attractive target for medicinal intervention. Retinoids (vitamin A and its natural metabolites and synthetic analogs) are currently the subject of intense biological interest stimulated by the discovery and characterization of retinoid receptor and the realization of these compounds as nonsteroidal small-molecule hormones [1, 2]. *All-trans*-retinoic acid (ATRA), the biologically most active metabolite of vitamin A, plays a major role in cellular differentiation and proliferation of epithelial tissue. ATRA is being used in differentiation therapy of cancer, in cancer chemoprevention and for the treatment of acne [3–5]. Recently, ATRA has proven useful in cancer chemotherapy [6–8]. One of the most impressive effects of ATRA is on acute promyelocytic leukaemia. Treatment of acute promyelocytic leukaemia patients with high dose of ATRA resulted in complete remission [9, 10]. Furthermore, several experiments in animals have demonstrated that ATRA inhibited the induction and caused the disappearance of prostate tumors [11]. In spite of these encouraging results, the effects of prolonged ATRA therapy on human cancers in the clinic has been scarce and disappointing [12]. It has been suggested that the therapeutic effects of ATRA are undermined by its rapid in vivo metabolism and catabolism by cytochrome P450 enzyme (CYPs) [13, 14].

One of the strategies for preventing in vivo catabolism of ATRA is to inhibit the P450 enzyme(s) responsible for this process. Indeed, this seems to be an emerging approach that may yield effective agents for the chemoprevention and/or treatment of cancers [15]. This may create a novel class of agents known as retinoic acid metabolism blocking agents (RAMBAs). Liarozole, a P-450 inhibitor (17, 20 steroid lyase) and the first RAMBA to undergo clinical investigation, preferentially increases intratumor levels of endogenous RA, resulting in antitumor activity [16]. This has opened up the possibility of developing more specific inhibitors of ATRA metabolism as a novel approach to cancer treatment. In the present study a wide range of established P450 inhibitors has been screened to examine their inhibitory activity on ATRA metabolism.

## Materials and Methods

### Reagents

*N*-benzyl-2-phenylethanamine was purchased from Aldrich Chemical Company Ltd, 2-methyl-5-phenyl-1,3-benzoxazole, 4-(4-bromophenyl)-1,2,3-thiadiazole, 5-(4-methyl-phenyl)-1,2,4-thiadiazole, 5-(3-chlorophenyl)-1,3-oxazole and 2-(thiophen-2-yl)-1,3,4-oxadiazole were obtained from Maybridge Chemical Co Ltd. (Tintagel Cornwall). Liarozole was donated by Janssen Research Foundation (Bearse Belgium). *All-trans-*retinoic acid, NADPH, butylated hydroxyanisole and all the other P-450 inhibitors were purchased from Sigma Chemical Company. [11,12-^3^H]-*All-trans-*retinoic acid (ATRA) was from DuPont (UK) Ltd. Formic acid, ammonium acetate and Hisafe III scintillation fluid (optiphase III) were obtained from Fisons Ltd. All solvents used for chromatography were of HPLC grade and were obtained from Rathburn Chemicals Ltd.UK. All other laboratory reagents were of analytical grade and obtained from British Drug House.

### Animals

Healthy male wistar rats were fasted overnight and killed by stunning.

### Preparation of rat liver microsomes

Rat liver microsomes were prepared by a previously described method [17] and stored at −80°C.

### RA Metabolism Assay

The incubation system contained RA (3 μM, μl), NADPH (2 mM, 50 μl), inhibitor (100 μM, final concentration) in DMSO (10 μl), phosphate buffer (50 mM, pH 7.4) up to a final volume of 400 μl. The reaction was started by the addition of male rat hepatic microsomes (0.12 mg/ml, 10 μl) and the mixture incubated at 37°C. The reaction was terminated after 15 min by addition of formic acid (1% 100 μl). ^3^H.RA and its metabolites were extracted into ethyl acetate containing 0.05% (v/v) butylated hydroxyanisole (2×2 ml). The extract was dried out in vacuum at r. t. and the residue dissolved in the mobile phase used for the reverse phase. Controls (10 μl, DMSO) were also carried with each experiment.

### HPLC Separation of labeled material

The assay followed a previously described one [17]: The column was a 10 μm C18 μ Bondapak (3.9× 300mm, Millipore) and the mobile phase was acetonitrile: water: formic acid (75:25:0.05v/v) containing 10 mM ammonium acetate. Flow rate was 1.2 ml.Min^−1^. Eluted ^3^H compounds were detected on line by a model 970 detector (Reeve) using Hisafe III scintillation fluid. The retention time of ^3^H-RA was 10 min and oxidative metabolites of ^3^H-RA were detected, eluting samples over 3 to 7 min. Metabolism was determined from the % conversion of RA into its total metabolites based on AUC values.

### Determination of IC50 values for potent P-450 inhibitors of retinoic acid metabolism

The IC50 values were determined for compounds exhibiting > 50% inhibition in the preliminary screening at 100 μM.

The incubations were performed in triplicate using varying inhibitor concentrations in DMSO (10 μl) (as determined by preliminary experiments), RA (3 μM, 10 μl), NADPH (2 mM, 50 ml), phosphate buffer (50 mM, pH 7.4, 320 μl) and male rat hepatic microsomes (0.12 mg/ml, 10 μl) at 37°C for 15 min. The reaction was terminated by addition of formic acid (1% 100μl), and HPLC analysis was performed by the method described above. Three separate experiments (each with triplicate tubes) were carried out.

## Results

### Preliminary screening of P450 inhibitors on the in vitro inhibition of RA metabolism

Forty-one known P450 inhibitors belonging to antifungal (mostly), analgesic, anticoagulant, anticonvulsants, antineoplastics, histamine, antihistamines and some miscellaneous drug classes were tested for their inhibitory activity against RA metabolism. Most of them are nitrogen-containing compounds. The results presented in table 1 showed that among these compounds only six compounds (*N*-benzyl-2-phenylethanamine, itraconazole, chlorpromazine, 5-chloro-1,3-benzoxazol-2-amine, proadifen and furazolidone) showed inhibition of RA metabolism which was > 50%. Ketoconazole and liarozole were also screened as standard potent inhibitors in the same system and gave 87.5% and 89% inhibition, respectively.

### IC50 values for Potent Inhibitors of RA Metabolism

The IC50 is the concentration of an inhibitor required to inhibit the enzyme by 50% at a given substrate concentration. IC50 were determined for compounds exhibiting > 50% inhibition in the preliminary screening at 100 μM. The IC50 for ketoconazole and liarozole were determined for comparative purposes.

**Fig. 1:**
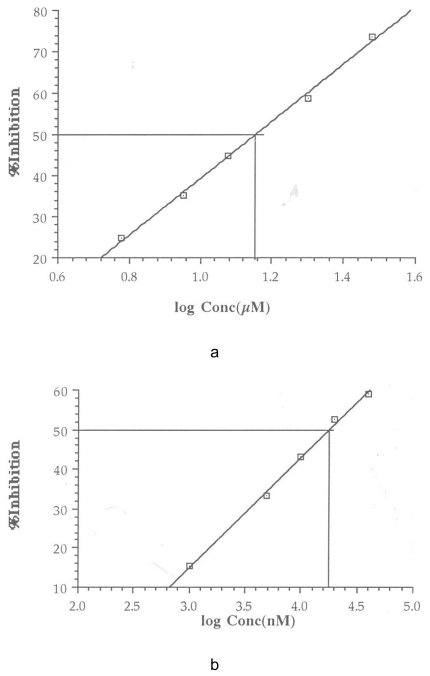
Determination of IC_50_ for ketoconazole (a) and itraconazole (b)

**Fig. 2:**
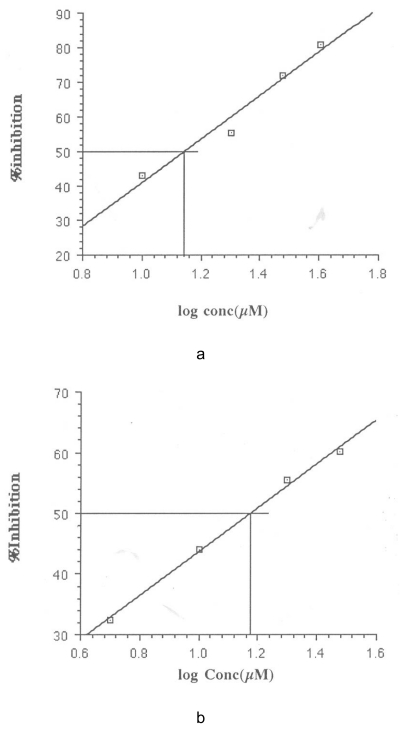
Determination of IC_50_ for *N*-benzyl-2-phenylethanamine (a) and Chlorpromazine (b)

**Fig. 3:**
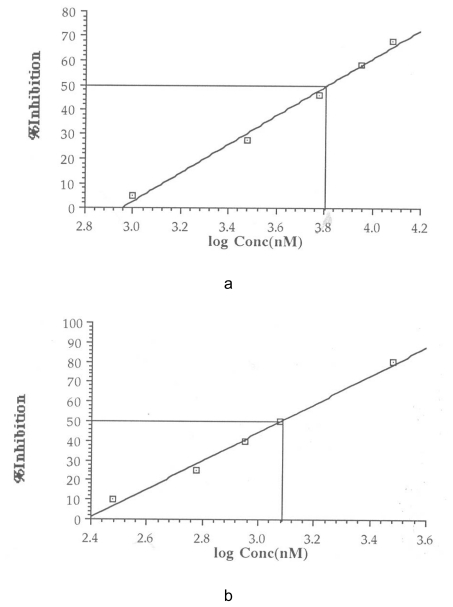
Determination of IC_50_ for 5-chloro-1,3-benzoxazol-2-amine (a) and Proadifen (b)

**Fig. 4:**
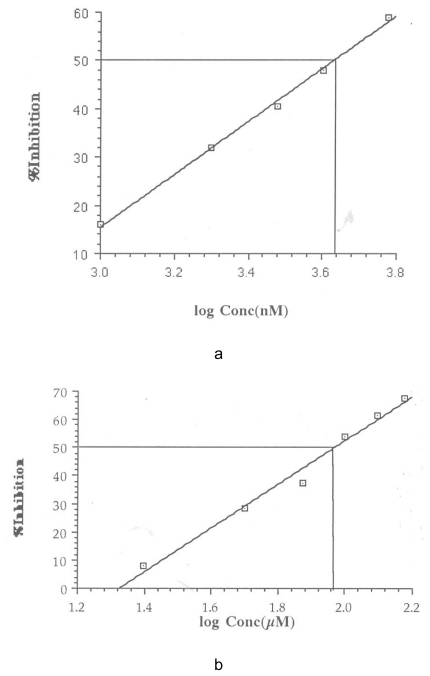
Determination of IC_50_ for Liarozole (a) and Furazolidone (b)

The IC50 was calculated from a plot of percentage inhibition versus log inhibitor concentration using Cricket Graph 1.3. These are shown in Fig 1–4. The IC50 values are summarized in table 2.

## Discussion

Retinoic acid (RA) concentrations either too low or too high (normal serum RA levels 0.5–7.0 ng/ml) adversely affect differentiation and maintenance of the tissues [18, 19]. Normal embryonic development also requires RA as a transcriptional regulator during specific times and at specific stages [20–22].

A dose of 10 mg/Kg body weight of RA has been shown to result in an approximately 50-fold increase in the level of RA in mouse embryo limb buds and generally results in developmental abnormalities. While a dose of 1 mg/Kg, which is approximately 20 times higher than the daily dose of RA necessary to support adequate growth of vitamin A-deficient animals produced no teratogenic effects in mice [23].

Either abnormal concentrations of RA (either higher or lower than normal) or transiently inappropriate RA availability cause central nervous system, limb and craniofacial defects in mice [23, 24]. Therefore, maintaining the correct RA concentrations demands tight control over RA biosynthesis and metabolism.

In contrast to retinal, which is stored in tissues and which displays stable plasma levels under regulation from the tissue stores, RA is rapidly metabolized in tissues and rapidly cleared from the plasma (t ½ < 1h) [25]. A cytochrome P450 (CYP) dependent oxidation in the 4 position of RA appears to be the rate limiting first step in the metabolism of RA to polar metabolites, the latter being biologically less active [25–27]. The significance of this catabolism of RA in relation to the biological and pharmacological effects of RA has received attention only recently. Warell and co-workers noted that patients who had received exogenous RA chronically to induce the remission of acute promelocytic leukemia (APL) later presented with increased plasma clearance of RA due to induction of metabolism [28, 29]. The enhanced plasma clearance of RA may have contributed to the failure of RA therapy to maintain remissions in these APL patients.

Liarozole fumarate is an imidazole cytchrome P450 inhibitor that has been shown to inhibit the metabolism of RA in in vitro tumour systems [30, 31]. Liarozole has shown significant antitumour effects in animal models and has clinical benefit in patients with advanced prostate cancers [32–34]. This anticancer activity owes part of its effectiveness to inhibition of the oxidative metabolism of RA with a concomitant elevation of tumour RA levels. This has opened up the possibility of developing more specific inhibitors of RA metabolism as a novel approach to cancer treatment. In the present investigation a wide range of established P450 inhibitors has been screened to examine their inhibitory activity on RA metabolism.

In the present studies different chemical ligands belonging to different chemical and pharmacological groups have been examined to study their possibility as RA metabolism inhibitor. The results indicate that mostly azoles with substituents in positions other than the 1-position on the ring are very weak inhibitors of RA metabolism. The most effective inhibitors (ketoconazole, itraconazole, bifonazole and clotrimazole) are 1-substituted and possess relatively large aromatic groups in the molecule. This reflects that 1-substituted imidazole bind to cytochrome P-450 with a very high affinity but substitution in the other position of the imidazole decreases the binding affinity.

Njar et al. [35], have explained that introduction of azole group at C-4 of ATRA may yield specific and potent inhibitors of ATRA-4-hydroxylase. Indeed they have described the synthesis of a number of novel 4 azolyl ATRA derivatives, some of which are amongst the most potent inhibitors of this enzyme [35].

Further study of these structure-activity relationships may provide useful lead compounds that could offer a novel approach to cancer treatment.

Although several cytochrome P450 enzymes (CYPs) have been shown to be involved in the catalysis of ATRA 4-hydroxylation, their specificity for ATRA is generally low [28, 36–39]. Recently, a new family of cytochrome P450 enzymes, CYP26A1, has been cloned and characterized in zebra fish, human, and mouse tissues [40]. CYP26A1 is ATRA-inducible and appears to be the most dedicated ATRA 4-hydroxylase enzyme known [40].

The compounds designed to inhibit CYP26A1 activity may be useful in elevating normal tissue ATRA levels or maintaining high therapeutic levels of ATRA. As such, it is an attractive pharmacological target for drug development when one aims to increase circulating or cellular RA concentrations. Further study of structure-activity relationships may provide useful lead compounds that could offer a novel approach to cancer treatment as does by liarozole for the treatment of prostate cancer [16 ].

## Figures and Tables

**Tab. 1:** In vitro inhibition of retinoic acid metabolism by P-450 inhibitors (100 μM). (Values are means of three determinations,(n=3), individual values differ from the mean by less than 3.1%. The symbol (-) denotes stimulation of RA metabolism).

**Antifungal Compounds**	

(a) *Imidazoles and related ring systems*	

Compound	% Inhibition

Liarozole	89.0
Ketoconazole	87.5
Itraconazole	68.7
Bifonazole	47.5
Clotrimazole	34.4
Econazole	14.0
Miconazole	13.5
1-Benzylimidazole	7.6
Sulconazole	8.7

(a) *Non imidazoles*	

Compound	% Inhibition

Griseofulvin	0.0
Amphotericin	6.24
Nystatin	5.44

*(c) Oxazoles and Thiazoles*	

Compound	% Inhibition

2-Methyl-5-phenyl-1,3-benzoxazole	18.0
4-(4-Bromophenyl)-1,2,3-thiadiazole	11.6
5-(4-Methylphenyl)-1,2,4-thiadiazole	4.8
5-(3-Chlorophenyl)-1,3-oxazole	8.6
2-(Thiophen-2-yl)-1,3,4-oxadiazole	0.0
5-Chloro-1,3-benzoxazol-2-amine	86.0

**Anthelmintic** (Azoles)	

Compound	% Inhibition

Tetramizole HCl	0.0
Thiabendazole	−4.31

**Anticoagulants** (Coumarin group)	

Compound	% Inhibition

4-Hydroxycoumarin	38.50
Coumarin	37.0
7-Methoxycoumarin	11.7
S-Warfarin	6.5
R-Warfarin	3.0

**Analgesics**	

Compound	% Inhibition

Aspirin	5.0
Paracetamol	3.5
Diflunisal	2.0
Indomethacin	0.0
Ketoprofen	−3.5

**Antineoplastic** (Aromatase inhibitors)	

Compound	% Inhibition

Aminoglutethimide	30.8
Nitroglutethimide	−1.4

**Anticonvulsants**	

Compound	% Inhibition

Nafimidone	12.0
Phensuximide	3.5

**Histamine and Antihistamines**	

Compound	% Inhibition

Thiopermide maleate	3.4
Cimetidine	1.0
Histamine	0.0

**Anorectics** (Benzphetamine and analogues)	

Compound	% Inhibition

*N*-Benzyl-2-phenylethanamine	85.0
Benzphetamine	46.0

**Miscellaneous Compounds**	

Compound	% Inhibition

Proadifen	86.0
Chlorpromazine	85.0
Furazolidone	54.0
Phenobarbitone	0.0

**Tab. 2:** IC50 values for some potent P-450 Inhibitors using male rat hepatic microsomes and retinoic acid as substrate (3μM).

Compound	IC50 (μM)
Proadifen	1.0 ± 0.14
Liarozole	4.2 ± 0.1
5-Chloro-1,3-benzoxazol-2-amine	6.5 ± 0.1
Ketoconazole	13.5 ± 1.3
Chlopromazine	16.9 ± 1.7
*N*-Benzyl-2-phenylethanamine	17.1 ± 3.2
Itraconazole	17.4 ± 1.0
Furazolidone	88.5 ± 6.0

Values are means ± S.D (n=3).

## References

[1] Evans RM (1988). The steroid and thyroid hormone receptor super family. Science.

[2] Katzenellenbogen JA, Katzenellenbogen BS (1996). Non-steroidal estrogen-receptor antagonists. Chem Biol.

[3] De Luca LM (1991). Retinoids and their receptors in differentiation, embryogenesis and neoplasia. FASEB J.

[4] Griffiths CEM, Fischer GJ, Finkel LJ, Voorhees JJ (1992). Mechanisms of action of retinoic acid in skin repair. Br J Dermatol.

[5] Lotan R (1996). Retinoids in cancer chemoprevention. FASEB J.

[6] Chopra DP, Wilkoff LJJ (1977). Reversal by Vit A analogues (Retinoids) of hyperplasia induced by N-methyl, N-nitro-N-nitrosoguanidine in mouse prostate organ cultures. J Natl Cancer Inst.

[7] Chytil F (1984). Retinoic acid: biochemistry, pharmacology, toxicology and therapeutic use. Pharmacol Rev.

[8] Lanitzki I, Goodman DS (1974). Inhibition of the effects of methylcholanthrene on mouse prostrate in organ culture by Vit A and its analogs. Cancer Res.

[9] Castaigne S, Chomienne C, Daniel MT, Ballerini P, Berger R, Fenaux P, Degos L (1990). All Trans retinoic acid as a differentiation therapy for acute promelocytic leukemia, I, clinical results. Blood.

[10] Huang ME, Ye YC, Chen SR, Chai JR, Lu JX, Zhoa L, Gu LJ, Wang ZY (1988). Use of All trans retinoic acid in the treatment of acute promelocytic leukemia. Blood.

[11] Hong WK, Itri L, Sporn MB, Roberts AB, Goodman DS (1994). Retinoid and human cancer. The Retinoids: Biology, Chemistry, and Medicine.

[12] Trump DL, Smith D, Stiff D, Adedoyin A, Bahnson R, Day R, Branch R (1994). All-trans-retinoic acid (ATRA) in hormone refractory prostate cancer (HPRC): ineffectiveness due to failure of drug delivery?. Proc Am Soc Clin Oncol.

[13] Muindi JR, Frankel SR, Huselton C, DeGrazia F, Garland WA, Young CW, Warrell RP (1992). Clinical pharmacology of oral All trans retinoic acid in patients with acute promelocytic leukemia. Cancer Res.

[14] Muindi J, Frankel SR, Miller WH, Jakubowski A, Scheinberg DA, Young CW, Dmitrovsky E, Warrell RP (1992). Continuous treatment with all Trans retinoic acid causes a progressive reduction in plasma drug concentrations. Implications for relapse and retinoid “resistance” in patients with acute promelocytic leukemia. Blood.

[15] Miller WH (1998). The emerging role of retinoids and retinoic acid metabolism blocking agents in the treatment of cancer. Cancer.

[16] Acevedo P, Bertram JS (1995). Liarozole potentiates the cancer chemopreventive activity of and the up-regulation of gap junctional communication and connexin 43 expression by retinoic acid and beta carotene in 10 T1/2 cells. Carcinogenesis.

[17] Ahmad M, Shabanah OA (2004). A comparative study on the in vitro hepatic metabolism of retinoic acid using different species. Sci Pharm.

[18] Fiorella PD, Napoli JL (1994). Microsomal retinoic acid metabolism. Effects of cellular retinoic acid binding protein (type1) and C-18 hydroxylation as an initial step. J Biol Chem.

[19] Napoli JL (1996). Retinoic acid biosynthesis and metabolism. FASEB J.

[20] Morriss-Kay GM (1992). Retinoic acid receptors in normal growth and development. Cancer Surveys.

[21] Morriss-Kay GM, Sokolova N (1996). Embryonic development and pattern formation. FASEB J.

[22] Summerbell D, Maden M (1990). Retinoic acid a developmental signaling molecule. Trends Neurosci.

[23] Harnish DC, Barua AB, Soprano KJ, Soprano DR (1990). Induction of beta retinoic acid receptor mRNA by teratogenic doses of retinoids in murine fetuses. Differentiation.

[24] Soprano DR, Harnish DC, Soprano KJ, Kochhar DM, Jiang H (1993). Correlations of RAR isoforms and cellular retinoid-binding proteins mRNA levels with retinoid induced teratogenesis. J Nutr.

[25] Duell AE, Astrom A, Griffiths CEM, Chambon P, Voorhes J (1992). Human skin levels of retinoic acid and cytochrome P-450 derived – 4-hydroxyl retinoic acid after topical application of retinoic acid in vivo compared to concentrations required to stimulate retinoic acid receptor mediated transcription in vitro. J Clin Invest.

[26] Njar VC (2002). Cytochrome P-450 retinoic acid 4 hydroxylase inhibitors: Potential agents for cancer therapy. Mini Rev Med Chem.

[27] Van Wauwe J, Coene MC, Cools W, Goossens J, Lauwers W, Jeune L, Hove C, Nyen G (1994). Liarozole fumarate inhibits the metabolism of keto- all- trans-retinoic acid. Biochem Pharmacol.

[28] Muindi JF, Young CW (1993). Lipid hydro peroxides greatly increase the rate of oxidative catabolism of all trans retinoic acid by human cell culture microsomes genetically enriched in specified cytochrome P-450 isoforms. Cancer Res.

[29] Warrell RP, DeThe H, Wang ZH, Degos LN (1991). Differentiation therapy of acute proxyelocytic leukemic with tretinoin (all Trans retinoic acid). Engl J Med.

[30] Wouters W, VanDon J, Dillen A, Coene MC (1992). Effects of liarozole, a new antitumoral compounds on retinoic acid induced inhibition of cell growth and on retinoic acid metabolism in MCF-7 human brest cancer cells. Cancer Res.

[31] Dijkman GA, Van Moorselar RJ, Van Ginckel R, Van Stratum P (1994). Antitumoral effects of liarozole in androgen dependent and independent R3327 Dunning prostate adenocarcinomas. J Urol.

[32] DeCoster R, Wouters W, Broynseels JJ (1996). P-450 dependent enzymes as targets for prostate cancer therapy. Steroid Biochem Mol Biol.

[33] Mahler C, Verhelst J, Denis J (1993). Ketoconazole and liarozole in the treatment of advanced prostatic cancer. Cancer.

[34] Van Ginckel R, Decoster R, Wouters W (1990). Antitumoral effects of R75251 on the growth of transplantable R3327 prostatic adencarcinoma in rats. Prostate.

[35] Njar VCO, Nnane IP, Brodie AMH (2000). Potent inhibition of retinoic acid metabolism enzyme(s) by novel azolyl retinoids. Bioorg Med Chem Lett.

[36] Leo MA, Lasker JM, Raucy JL, Kim C-I, Black M, Lieber CS (1989). Metabolism of retinol and retinoic acid by human liver cytochrome P 450 llC8. Arch Biochem Biophys.

[37] Nadin L, Murray M (1999). Participation of CYP 2C8 in retinoic acid 4 hydroxylation in human hepatic microsomes. Biochem Pharmacol.

[38] Njar VC, Gediya L, Purushottamachar P, Chopra P, Vasaitis TS, Khandelwal A, Mehta J, Huynh C, Belosay A, Patel J (2006). Retinoic acid metabolism blocking agents (RAMSAs) for treatment of cancer and dermatological diseases. Bioorg Med Chem.

[39] McCaffery P, Simons C (2007). Prospective teratology of retinoic acid metabolic blocking agents (RAMBAs) and loss of CYP26 activity. Curr Pharm Des.

[40] Thatcher JE, Isoherranen N (2009). The role of CYP26 enzymes in retinoic acid clearance. Expert Opin Drug Metab Toxicol.

